# Association of *STAT4* Polymorphisms with Susceptibility to Type-1 Autoimmune Hepatitis in the Japanese Population

**DOI:** 10.1371/journal.pone.0071382

**Published:** 2013-08-22

**Authors:** Kiyoshi Migita, Minoru Nakamura, Seigo Abiru, Yuka Jiuchi, Shinya Nagaoka, Atsumasa Komori, Satoru Hashimoto, Shigemune Bekki, Kazumi Yamasaki, Tatsuji Komatsu, Masaaki Shimada, Hiroshi Kouno, Taizo Hijioka, Motoyuki Kohjima, Makoto Nakamuta, Michio Kato, Kaname Yoshizawa, Hajime Ohta, Yoko Nakamura, Eiichi Takezaki, Hideo Nishimura, Takeaki Sato, Keisuke Ario, Noboru Hirashima, Yukio Oohara, Atsushi Naganuma, Toyokichi Muro, Hironori Sakai, Eiji Mita, Kazuhiro Sugi, Haruhiro Yamashita, Fujio Makita, Hiroshi Yatsuhashi, Hiromi Ishibashi, Michio Yasunami

**Affiliations:** 1 NHO-AIH Study Group, Nagasaki Medical Center, Omura, Nagasaki, Japan; 2 Department of Hepatology, Nagasaki University Graduate School of Biomedical Sciences, Nagasaki, Japan; 3 Department of Clinical Medicine, Institute of Tropical Medicine, Nagasaki University, Nagasaki, Japan; Institute of Medical Research A Lanari-IDIM, University of Buenos Aires-National Council of Scientific and Technological Research (CONICET), Argentina

## Abstract

**Background/Aims:**

Recent studies demonstrated an association of *STAT4* polymorphisms with autoimmune diseases including systemic lupus erythematosus and rheumatoid arthritis, indicating multiple autoimmune diseases share common susceptibility genes. We therefore investigated the influence of *STAT4* polymorphisms on the susceptibility and phenotype of type-1 autoimmune hepatitis in a Japanese National Hospital Organization (NHO) AIH multicenter cohort study.

**Methodology/Principal Findings:**

Genomic DNA from 460 individuals of Japanese origin including 230 patients with type-1 autoimmune hepatitis and 230 healthy controls was analyzed for two single nucleotide polymorphisms in the *STAT4* gene (rs7574865, rs7582694). The *STAT4* rs7574865T allele conferred risk for type-1 autoimmune hepatitis (OR = 1.61, 95% CI = 1.23–2.11; *P* = 0.001), and patients without accompanying autoimmune diseases exhibited an association with the rs7574865T allele (OR = 1.50, 95%CI = 1.13–1.99; *P* = 0.005). Detailed genotype-phenotype analysis of type-1 autoimmune hepatitis patients with (n = 44) or without liver cirrhosis (n = 186) demonstrated that rs7574865 was not associated with the development of liver cirrhosis and phenotype (biochemical data and the presence of auto-antibodies).

**Conclusions/Significance:**

This is the first study to show a positive association between a *STAT4* polymorphism and type-1 autoimmune hepatitis, suggesting that autoimmune hepatitis shares a gene commonly associated with risk for other autoimmune diseases.

## Introduction

Autoimmune hepatitis (AIH) is characterized by chronic inflammation of the liver, interface hepatitis, hypergammaglobulinemia and production of autoantibodies [1], [2]. The etiology of AIH is unknown, but is thought to have both a genetic and an environmental basis [3]. Although the HLA DRB1 gene is a well-characterized susceptibility gene [4], [5], non-HLA susceptibility genes may also contribute to genetic susceptibility to AIH and remain to be elucidated. Recently, with the emergence of genome-wide association studies (GWAS), there has been a dramatic increase in genetic discoveries for many complex genetic autoimmune diseases, such as type 1 diabetes and rheumatoid arthritis (RA) [6]. It is also interesting to note that evaluating the results from the study of one disease in other complex diseases can disclose common risk factors. Thus, there has been a marked overlap of loci between autoimmune diseases [7]. Of those, *STAT4* particularly has been confirmed in several studies and is clearly associated with autoimmune diseases such as RA or systemic lupus erythematosus (SLE) [8]–[10]. STAT4, a signal transducer and activator of transcription 4, is expressed in activated peripheral blood monocytes, dendritic cells and macrophages at the sites of inflammation in humans [11]. It is activated by interleukin (IL)-12, leading to T helper (Th) 1 and Th 17 differentiation, monocyte activation and interferon (IFN)-α production [12]. Since Th1 and Th17 cells have the capacity to cause autoimmunity [13], STAT4 may play a crucial role in the development of autoimmune diseases, including AIH.

The degree of risk for RA or SLE susceptibility observed with the *STAT4* haplotype was found to be similar in Caucasian and Japanese populations [14]–[16]. In addition, meta-analysis demonstrated that the *STAT4* rs7574865 T allele conferred susceptibility to various autoimmune diseases, suggesting an association between *STAT4* gene polymorphism and autoimmune diseases [17].

STAT4 is considered important in a mouse model of Th1-dependent liver injury [18]. Therefore, we hypothesized that *STAT4* polymorphisms may overlap in genetic susceptibility between AIH and other autoimmune diseases. To test this hypothesis, we investigated the association of *STAT4* with type-1 AIH susceptibility using a large series of Japan NHO-AIH registry [19]. We also tried to evaluate whether the gene was associated with type-1 AIH outcome measures in a Japanese AIH cohort.

## Materials and Methods

### Study population

Consecutive type-1 AIH patients were initially enrolled in the register of the Japanese National Hospital Organization (NHO) liver-network study, contributed to medical facilities in Japan, and prospectively followed since 2009 as a multicenter cohort population. All patients satisfied the 1999 revised criteria of International Autoimmune Hepatitis Group (IAIHG) diagnosis of type-1 AIH [20]. Patients were excluded from the study if there was histological evidence of cholangitis or non-alcoholic steatohepatitis. In addition, patients who were positive for hepatitis B virus (HBV)-surface antigen (HBsAg) or hepatitis C virus (HCV)-RNA were excluded. Patients with other causes of liver disease, such as excess alcohol or drug use, were excluded based on reviews of their appropriate history and investigations. The control group consisted of 230 gender-matched Japanese healthy subjects (34 men and 196 women). The mean ± SD age was 43.9±13.1 years. Among the cases (AIH) and controls, 156 patients and 163 controls were recruited from West Japan and 74 patients and 67 controls were recruited from East Japan. The study was approved by the Ethics committee of the Nagasaki Medical Center and participating NHO Liver-network hospitals ((NHO Sagamihara National Hospital, Tokyo National Hospital, Yokohama Medical Center, Nagoya Medical Center, Kure Medical Center, Osaka Minami Medical Center, Kyushu Medical Center, Minami Wakayama Medical Center, Shinshu Ueda Medical Center, Kanazawa Medical Center, Higashi Hiroshima Medical Center, Asahikawa Medical Center, Kokura Medical Center, Ureshino Medical Center, Higashi Nagoya National Hospital, Hokkaido Medical Center, Okayama Medical Center, Takasaki General Medical Center, Oita Medical Center, Beppu Medical Center, Osaka Medical Center, Kumamoto Medical Center, Nishigunma National Hospital). Written informed consent was obtained from each individual. This study was conducted with the approval of the ethical committees of Nagasaki Medical Center and participating NHO Liver-network hospitals. Written informed consent was obtained from each individual.

### Variables at study entry

Demographic and other characteristics of the 230 retained patients were recorded in a database at the initial assessment. Data included sex, age at diagnosis, time of onset of symptoms or other evidence of liver disease, markers of infection with hepatitis viruses HBV and HCV, alcohol intake, coexisting autoimmune diseases, serum levels of ALT, AST, alkaline phosphatase and bilirubin, platelet count and prothrombin time. Anti-nuclear antibodies (ANA) and anti-smooth muscle antibodies (ASMA) were measured by indirect immunofluorescence on HEp-2 cells and cut-off titers for positivity were 1∶40. Liver tissue from percutaneous biopsy performed at the referring facility was available for the majority of patients at the time of entry (192/230, 83.5%), but for only a few at the subsequent follow-up examination (7/230, 3.0%). The histological variables examined included degree of fibrosis (0; absent, 1; expansion of fibrosis to parenchyma, 2; portal-central or portal-portal bridging fibrosis, 3; presence of numerous fibrous septa, 4; multi-nodular cirrhosis). The histological diagnosis of cirrhosis required a loss of the normal lobular architecture, reconstruction of hepatic nodules and presence of regenerative nodules [21]. Liver biopsy was not performed for patients who had apparent biochemical, endoscopic and ultrasound features of liver cirrhosis. All phenotypic data were collected blind to the results of the genotypic data.

### DNA extraction and genotyping

Blood samples were taken from all study participants, and genomic DNA was isolated from peripheral blood leukocytes using a DNA blood mini kit from Qiagen (Hilden, Germany) according to the manufacturer's guidelines. STAT4 SNPs (rs7574865, rs7582694) were determined by the polymerase chain reaction–restriction fragment length polymorphism (PCR–RFLP) method [22], [23]. The primers used for the PCR reaction were rs7574865, F:5′-AAAGAAGTGGGATAAAAAGAAGTTTG-3′, R:5′-CCACTGAAATAAGATAACCACTGT-3′, and rs7582694, F:5′-ATCCAACTCTTCTCAGCCCTT-3′, R:5′-TCATAATCAGGAGAGAGGAGT-3′.

Rs7574865 was a 147-bp PCR product and was digested with restriction enzyme *Hpa*I (New England Biolabs) and electrophoresed on a 2.5% polyacrylamide gel. Rs7574865 was a 338-bp PCR product was digested with restriction enzyme *Hpy*CH4III (New England Biolabs) and electrophoresed on a 3.0% polyacrylamide gel.

HLA-DRB1 genotyping was performed as described previously [24]. Briefly, the HLA-DRB1 genotype was determined by sequence-based typing (SBT) of group-specific PCR products.

### Statistical analyses

Results are expressed as mean ± SD. The statistical significance of differences between groups was calculated by either the chi-square test or Fisher's exact test for categorical data and Mann-Whitney's *U*-test for quantitative data. Multivariate logistic regression analysis was performed with SPSS v.18 for windows (SPSS Statistics, Illinois). Deviation from Hardy-Weinberg equilibrium was assessed using the SNPAlyze software ver. 7.0 (Dynacom, Yokohama, Japan). Power calculations were performed by using an online power calculator [25]. A *P* value of <0.05 was considered significant.

## Results

### Baseline data at entry

Of the original 240 patients registered in the NHO-AIH study, 10 were excluded from analysis because of overlapping primary biliary cirrhosis (PBC). The remaining 230 patients were eligible for the study. Table 1 shows other demographic data for the cohort at entry. Among the enrolled type-1 AIH patients, 206 (89.6%) were positive for ANA (>1∶40) and 96 (41.7%) for ASMA (>1∶40). Some patients with lower serum aminotransferase or total bilirubin were managed with ursodeoxycholic acid (UDCA) therapy alone, which was demonstrated to be efficacious in Japanese patients with type I autoimmune hepatitis [26]. Among 230 eligible patients, 29 (12.6%) had liver cirrhosis at the time of diagnosis, and among the remaining 201 patients without liver cirrhosis, 15 developed liver cirrhosis during the follow-up. Two patients died because of complications (ruptured esophageal varices 1, hepatic failure 1) of liver cirrhosis during follow-up.

**Table 1:** Baseline characteristics of type-1 AIH patients.

	n = 230
Gender (male/female)	23/207
Age at presentaion (years)	59.6±12.2
Other autoimune diseases	39(17.0%)
Baseline Laboratory Values	
AST (<40 IU/L)	432.5±444.1
ALT (<40 IU/L)	484.3±490.5
ALP (<112 IU/L)	463.5±210.3
Total Bilirubin (mg/ml)	3.83±6.14
Albumin (3.5–5.0 g/L)	3.85±0.67
IgG (870–1700 mg/dl)	2489.4±931.4
Platelets (15–40×10^4^/μl)	18.6±7.1
ANA + (≥1:40)	206(89.6%)
ASMA + (≥1:40)	96(41.7%)
Cirrhosis at presentation	44(19.1%)
Received treatment	
Steroid alone	81(35.2%)
Steroid + UDCA	72(31.3%)
Steroid + Aza	15(6.5%)
UDCA alone	49(21.3%)

Abbreviations: AIH; autoimmune hepatitis, AST; aspartate aminotransferase, ALT; alanine aminotransferase, ALP; alkaline phosphate, IgG; immunoglobulin G, ANA; anti-nuclear antibody, ASMA; anti-smooth muscle antibody, UDCA; ursodeoxy cholic acid, Aza; azathioprine. Data are expressed as number (percentage) or mean ± standard deviations.

### Association of *STAT4* polymorphisms with type-1 AIH

The genotype frequencies for *STAT4* rs7574865 and rs7582694 were in HWE (Hardy-Weinberg equilibrium) in both the patient and control populations (data not shown). Because of the strong linkage disequilibrium between rs7574865 and rs7582694 (R^2^ = 0.949 and D' = 0.981), very similar results were observed between rs7574865 (Table 2) and rs7582694 (Table 3). We observed a significant difference in allele frequency and genotype distribution of *STAT4* polymorphisms (rs7574865) between type-1 AIH patients and controls. As shown in Table 2, the minor T allele and TT genotype frequencies at *STAT4* rs7574865 in the type-1 AIH group differed significantly from those in the control group.

**Table 2:** STAT4 rs7574865 polymorphism in patients with type-1 AIH and controls.

		Control (%)	AIH (%)	*p*-value^a^	OR (95%CI)
		n = 230	n = 230		
Genotype frequencies				0.001	
	G/G	103(44.8)	77(33.5)		
	G/T	108(47.0)	109(47.4)		
	T/T	19(8.3)	44(19.1)		
Allele				0.001	
	G	314(68.3)	263(57.2)		1
	T	146(31.7)	197(42.8)		1.611(1.230–2.109)

Abbreviation: AIH; autoimmune hepatitis, OR; odds ratio, CI; confidence interval, STAT4; signal transducer and activator or transcription.

a Genotype frequencies were determined by χ2 test using 2×3 contingency tables between patients with AIH and healthy controls. Allele frequencies were determined by χ2 test using 2×2 contingency tables between patients with AIH and healthy controls.

**Table 3:** STAT4 rs7582694 polymorphism in patients with type-1 AIH and controls.

		Control (%)	AIH (%)	*p*-value^a^	OR (95%CI)
		n = 230	n = 230		
Genotype frequencies				0.001	
	G/G	101(43.9)	80(34.8)		
	G/C	109(47.4)	103(44.8)		
	C/C	20(8.7)	47(20.4)		
Allele				0.001	
	G	311(67.6)	263(57.2)		1
	C	149(32.4)	197(42.8)		1.563(1.195–2.046)

Abbreviation: AIH; autoimmune hepatitis, OR; odds ratio, CI; confidence interval, STAT4; signal transducer and activator or transcription.

a Genotype frequencies were determined by χ2 test using 2×3 contingency tables between patients with AIH and healthy controls. Allele frequencies were determined by χ2 test using 2×2 contingency tables between patients with AIH and healthy controls.

To determine whether the observed association of the *STAT4* gene SNPs with disease susceptibility was caused by other autoimmune diseases associated with AIH, we stratified type-1 AIH patients without other overlapping autoimmune diseases. There was a significant association of *STAT4* rs7574865 with susceptibility to type-1 AIH even in the AIH patients without other overlapping autoimmune diseases (Table 4).

**Table 4:** STAT4 rs7574865 polymorphism in patients with type-1 AIH without other autoimmune diseases.

		Control (%)	AIH without other autoimmune diseases (%)	*p*-value^a^	OR (95%CI)
		n = 230	n = 191		
Genotype frequencies				0.008	
	G/G	103(44.8)	68(35.6)		
	G/T	108(47.0)	89(46.6)		
	T/T	19(8.3)	34(17.8)		
Allele				0.005	
	G	314(68.3)	225(58.9)		1
	T	146(31.7)	157(41.1)		1.501(1.131–1.992)

Abbreviation: AIH; autoimmune hepatitis, OR; odds ratio, CI; confidence interval, STAT4; signal transducer and activator or transcription.

a Genotype frequencies were determined by χ2 test using 2×3 contingency tables between patients with AIH and healthy controls. Allele frequencies were determined by χ2 test using 2×2 contingency tables between patients with AIH and healthy controls.

### Associations between *STAT4* genotype status and type-1AIH phenotype

To examine the associations between HLA-DR and type-1 AIH, HLA-DR allele typing was performed in patients with type-1 AIH. In the analysis of HLA-DR alleles, the frequencies of DR *04 allele was significantly increased in type-1 AIH patients as compared with those in controls (Table 5). The STAT4 rs7574865 T allele and HLA-DR *04 allele for the progression to liver cirrhosis were subjected to multivariate logistic regression analysis. Neither HLA-DR *04 allele nor rs7574865 T allele did not contribute to the progression to liver cirrhosis (data not shown). Based on the significant association of the rs7574865 with susceptibility to type-1 AIH, we also performed a detailed genotype-phenotype analysis using the clinical data. However, we found no significant difference in the presence of autoantibodies (ANA or ASMA) and the peak levels of transaminases or total bilirubin (AST, ALT, TB) by laboratory tests among each genotype (data not shown).

**Table 5:** Distribution of HLA-DR alleles distribution in patients with type-1 AIH.

*HAL-DR alleles*	AIH	Control	*P*	*Pc*	OR (95%CI)
	Alleles, No.(%)	Alleles, No.(%)			
	(n = 460 alleles)	(n = 460 alleles)			
*01	8(1.7)	24(5.2)	0.004	0.052	0.322(0.143–0.723)
*04	189(41.1)	118(25.7)	0.000001	0.000013	2.021(1.528–2.674)
*07	1(0.2)	4(0.9)	0.187	2.431	0.248(0.028–2.231)
*08	67(14.6)	42(9.1)	0.011	0.143	1.697(1.126–2.556)
*09	52(11.3)	70(15.2)	0.080	1.040	0.710(0.483–1.043)
*10	4(0.9)	2(0.4)	0.343	4.459	2.009(0.366–11.021)
*11	7(1.5)	7(1.5)	1.000	13.000	1.000(0.348–2.874)
*12	19(4.1)	26(5.7)	0.285	3.705	0.719(0.392–1.319)
*13	16(3.5)	47(10.2)	0.000052	0.000676	0.317(0.177–0.567)
*14	26(5.7)	28(6.1)	0.779	10.127	0.924(0.533–1.602)
*15	66(14.3)	88(19.1)	0.052	0.676	0.708(0.499–1.004)
*16	4(0.9)	2(0.4)	0.343	4.459	2.009(0.366–11.021)
*17	1(0.2)	2(0.4)	0.500	6.500	0.499(0.045–5.521)

HLA-DRB1 allele was assessed by cis-square test. The probability values were corrected (*Pc*) for multiple testing (Bonferroni correction).

## Discussion

AIH reflects a complex interaction between triggering factors, environmental factors, genetic predisposition and the immune regulatory network [3]. Most knowledge concerning the genetic factors of AIH comes from studies of the HLA genes [4], [5]. Although multiple genes are probably involved, HLA genes appear to play a dominant role in the predisposition to AIH [27]. Genetic factors other than HLA genes that can affect the susceptibility of AIH are mainly polymorphisms in genes that encode proteins that affect cytokine pathways responsible for modulating immunity [27]–[29]. Although autoimmune diseases include a wide array of different organ involvement and symptoms, they all share a common component: the loss of immune tolerance toward “self antigen” [30]. Findings in recent genetic studies support the emerging concept that distinct clinical autoimmune diseases may share genetic susceptibility factors. STAT4 is a critical transcription factor involved in the regulation of Th1/Th2 cytokine balance [12]. *STAT4* polymorphisms have been found to be associated with various autoimmune diseases [8]–[10].

This study is the first to investigate a detailed correlation between *STAT4* gene polymorphisms and susceptibility to type-1 AIH in a Japanese nationwide AIH cohort study. In the current study, we confirmed an association of *STAT4* polymorphisms with susceptibility to type-1 AIH. Our data suggest that *STAT4* may be an “autoimmune disease susceptibility gene” and support the concept of deregulated pathways across multiple autoimmune diseases. In addition to their influence on autoimmune disease susceptibility, *STAT4* polymorphisms can also influence disease phenotypes. For example, rs7574865 in SLE patients was associated with severe disease manifestations, such as nephritis, high double stranded-DNA antibody production and younger age of disease onset. [31] For patients with systemic sclerosis, this polymorphism was associated with the presence of pulmonary fibrosis [32]. Therefore, we examined possible associations between *STAT4* and the clinical phenotype of type-1 AIH. However, we did not find evidence of association between *STAT4* polymorphisms and disease progression or phenotype of type-1 AIH.

Regarding the disease-developing effect of genetic variants in the *STAT4* region on type-1 AIH observed in our study, it might be interesting to determine whether the *STAT4* risk alleles have different expression levels or functional effects in different effector cells [33]. The susceptibility SNP rs7574865 is located within intron 3 of *STAT4*, a non-coding region. It is suspected that it may influence the gene expression of STAT4 at the level of transcription or splicing variation [34]. A recent study reported that the expression level of STAT4 in peripheral blood mononuclear cells correlated with the risk allele of STAT4 rs7574865 [33]. This might indicate the effects of different *STAT4* gene variants on STAT4 expression levels. To date, the main alternative spliced isoforms of *STAT4* are STAT4α and STAT4β. STAT4β is a shorter form of the full-length STAT4α and is not as efficient as STAT4α for the direct induction of IFN-γ gene expression activated by IL-12 in Th1 cells [35]. However, expression of STAT4β, lacking the transactivation domain, was not affected by the STAT4 SNPs [33]. Additionally, a significant inverse correlation with T-risk alleles at rs7574865 and the methylation status of the *STAT4* promoter was demonstrated in inflammatory bowel disease [36]. The *STAT1* gene is located adjacent to *STAT4* suggesting it is also a candidate susceptibility gene for autoimmune disease [37]. To examine the role of the STAT1-STAT4 region, 52 tag SNPs encompassing this region in Japanese lupus patients [38]. The SNPs rs11889341 and rs10168266 were in linkage disequilibrium (LD) with rs7574865 and were significantly associated with SLE [38]. In contrast, significant association was not detected for SNPs in the *STAT1* region [38].

AIH pathogenesis are more complex than the traditional dichotomous Th1/Th2 paradigm, where STAT4 represents a transcription factor that induces IL-12, IL-23 and type 1 IFN-mediated signals to Th1 and Th17 differentiation, monocyte activation and interferon-γ production [39]. STAT4 is important for IL-22 production, which plays a pathological role in IL-17-dependent hepatitis [40].

A recent study showed that G allele at rs7574865 was associated with increased risk for HCC, suggesting dual roles of STAT4 in autoimmune diseases and HBV-related HCC [41]. Interestingly, subjects with GG genotype at rs7574865 had the lowest mRNA levels of *STAT4* in both HCC and non-tumor tissues compared with TG and TT genotypes [41]. Considering the role of STAT4 in Th1 immune responses, rs7574865 polymorphisms may affect the hepatic immune response against auto-antigen or viral antigen, contributing to the susceptibility of these related disorders. Further studies will be needed to examine the different possible mechanisms by which the variant haplotypes contribute to AIH.

The current study was limited because there were relatively small numbers of patients, and because some of the phenotypes examined were related to disease activity, and therefore may have fluctuated naturally or as a result of treatment. Additionally, it was difficult to perform a replication study due to the very low prevalence of type-1 autoimmune hepatitis and limited numbers of enrolled patients. In the current study, the power to detect a 1.6-fold increased risk, assuming an alpha value of 0.05, was 0.627 for rs7574865 T allele. Another limitation is the lack of complete information regarding the causal polymorphisms and their exact functional roles.

In summary, our results identified *STAT4* SNP rs7574865 as a disease-susceptible gene variant in type-1 AIH. Further studies on the expression and regulation of *STAT4* in the liver will be required to investigate the functional consequences of *STAT4* gene variants in more detail.
